# Experimental verification of a 3D *in vivo* dose monitoring system based on EPID

**DOI:** 10.18632/oncotarget.22758

**Published:** 2017-11-30

**Authors:** Xiaoyong Wang, Lixin Chen, Conghua Xie, Dajiang Wang, Gaili Chen, Zhengming Fu, Hui Liu

**Affiliations:** ^1^ Department of Radiation and Medical Oncology, Zhongnan Hospital of Wuhan University, 430071 Wuhan, China; ^2^ Sun Yat-sen University Cancer Center, State Key Laboratory of Oncology in South China, Collaborative Innovation Center for Cancer Medicine, 510060 Guangzhou, China; ^3^ Cancer Center, Renmin Hospital of Wuhan University, 430060 Wuhan, China

**Keywords:** electronic portal imaging device (EPID), 3D dose reconstruction, *in vivo*

## Abstract

**Purpose:**

To evaluate the Edose system, a novel three-dimensional (3D) *in vivo* dose monitoring system based on electronic portal imaging device (EPID), prior to clinical application, we analyzed the preliminary clinical data using Edose system in patients receiving intensity-modulated radiation therapy (IMRT).

**Materials and methods:**

After the physical modeling, the measured results from the Edose system were examined in homogeneous and inhomogeneous phantoms, respectively. To verify the accuracy of the Edose system, we compared its results with testing results from ionization chamber, measurement matrix (Delta4) and dosimetric films. The dosimetric performance of the Edose system was evaluated in 12 randomly selected patients with IMRT and VMAT, and the measured results were compared with the treatment plans.

**Results:**

Compared with the measured results, the dose difference at the center of target volume was (0.12±0.91)% and (0.03±0.85)%, the γ pass rate was (94.18±1.69)% and (95.24±1.62)% (3mm/3%)for homogeneous and inhomogeneous phantoms, respectively. For IMRT patients, the dose difference at the center of target volume was (0.75±1.53)%, and the γ pass rates were (89.11±3.24)% (3mm/3%) and (96.40±1.47)% (3mm/5%), respectively. Compared with the results of DVH, the maximum differences of PTVs and mostly organs at risk were all within 3%. For VMAT patients, the γ pass rates were (93.04 ± 2.62)% (3mm/3%) and (97.92 ± 1.38)% (3mm/5%), respectively.

**Conclusions:**

*In vivo* dose monitoring may further improve the safety and quality assurance for radiation therapy. But rigorous clinical testing is required before putting the existing commercial systems into clinical application. In addition, more clinical experiences and better workflows for using the Edose system are needed.

## INTRODUCTION

With the rapid progress of the radiation therapy techniques such as intensity-modulated radiation therapy (IMRT) and stereotactic body radiation therapy (SBRT), the radiation treatment delivery becomes more complex. This brings more uncertainties and variations in the treatment process such as gantry rotation speed, collimator angle, dose rate, moving speed and position accuracy of multi-leaf collimator (MLC), etc. [1, 2]. All these factors can result in the real radiation dose deviating from the original planned dose. These errors, if severe, may even lead to radiotherapy accidents [3]. Therefore, radiation dose verification has become one of the top clinical priorities in the quality assurances for modern radiotherapy.

Currently, the most common method for dose verification of radiotherapy is pre-treatment dose verification in homogeneous phantoms using detector matrix and films [4-8]. However, given the fact that there are big differences between the homogeneous phantom and the human body, how well the measurement results is associated with the actual dosage received by the target volume or organs at risk is a concern; In addition, there are also defects to determine how accurately the treatment plan is performed as planned only by γ pass rate [9]. More importantly, pre-treatment dose verification may only ensure the accuracy of plan implementation by the accelerator; it may not truly ensure the actual dose patients received during the treatment [10-12]. Thus, more and more attentions have been paid to *in vivo* dose monitoring [13], especially at the modern radiotherapy era.

Given the high resolution and ease to use of EPID, at present, more and more researches have focused on the *in vivo* dose verification based on EPID [14-20]. Some software such as “Dosimetry check” had been gradually utilized in clinical practice [21]. There were reports showing that *in vivo* dose verification using EPID can effectively reduce the dosimetric errors in radiotherapy [11]. Those *in vivo* dose verification systems which based on EPID commonly require appropriate dosimetric algorithms matching the systems. Current methods mainly include: i) Use the “back-projection” method to reconstruct the dose distribution of patients according to actual measurement results by EPID; ii) Backstepping fluence maps according to actual EPID measurements, then using calculation model such as collapsed-cone convolution (CCC) algorithm, Pencil-beam calculation (PBC) algorithm, etc. to reconstruct the 3D dose distribution for patients.

In this study, we carried out 3D *in vivo* dose monitoring in clinic by using a novel commercial 3D dose monitoring system (Edose 3.02, Company of Raydose, China) based on EPID. It includes mainly two aspects: 1) After the physical modeling for this system, preclinical tests were carried out; 2) Preliminary analysis for clinical application was done for actual patients. Through clinical testing, we can preliminarily assess the accuracy for dose monitoring using this commercial system. And we can explore better clinical workflow as clinical experience accumulated.

## RESULTS

### Results of physical modeling

**Figure 1:**
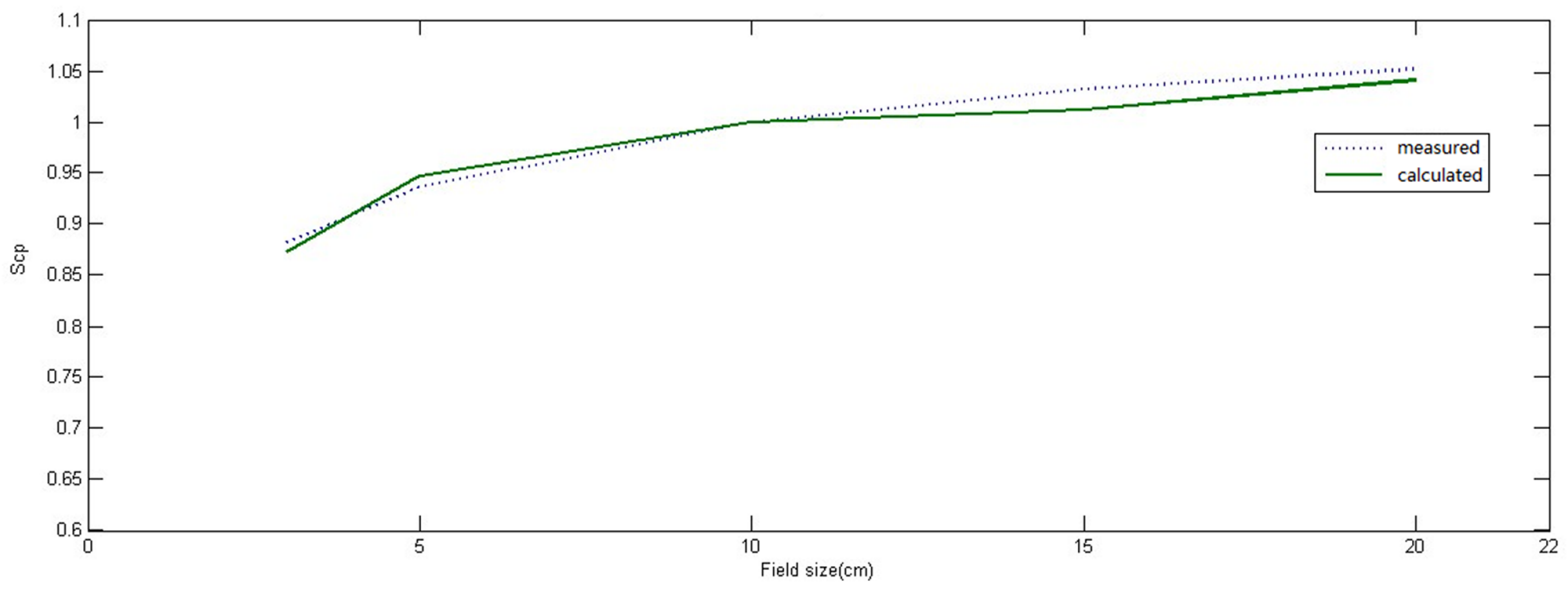
Comparison between output factor (Sc, p) of measured radiation field in three-dimensional water tank and reconstructed value of the physical model in different radiation field (normalization to 10 cm × 10 cm radiation field) The solid line represented calculated values of physical model, the dotted line represented measured values in the three-dimensional water tank.

**Figure 2:**
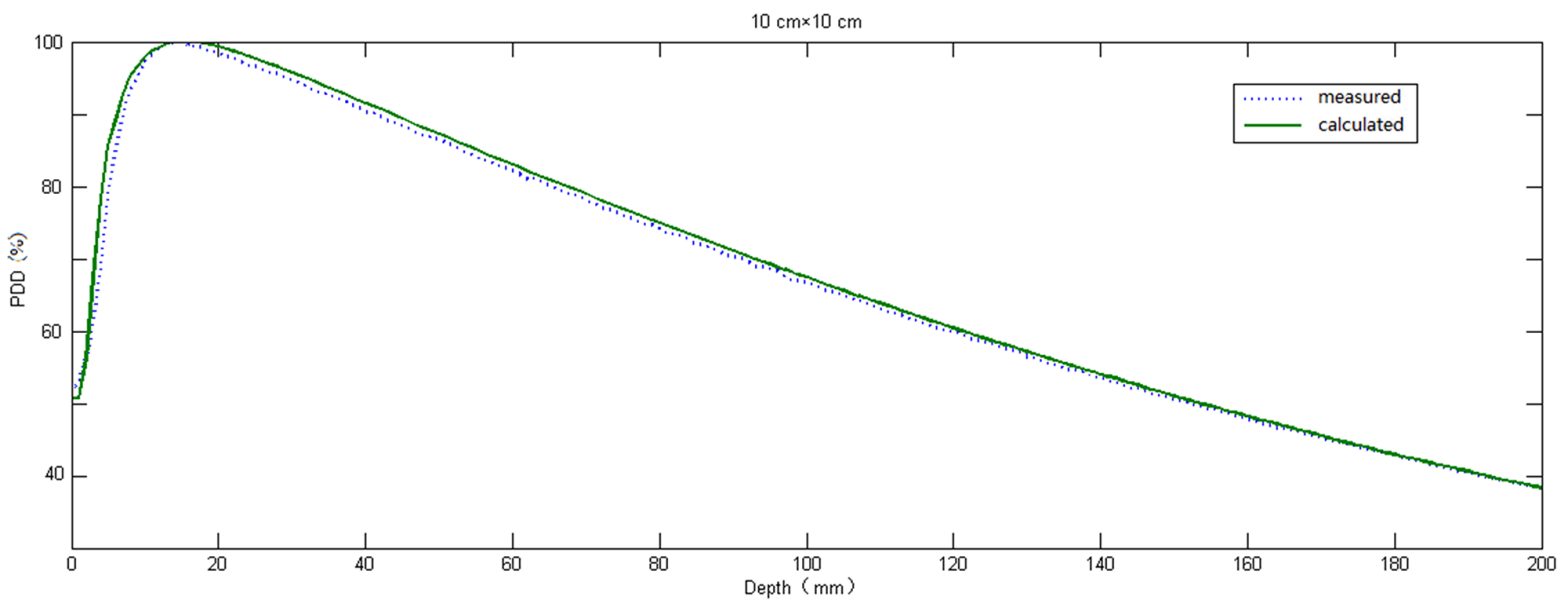
Comparison between PDD measured in the three-dimensional water tank and reconstructed value of the physical model in 10 cm × 10 cm radiation field The solid line represented calculated values of physical model, the dotted line represent measured values in the three-dimensional water tank.

**Figure 3:**
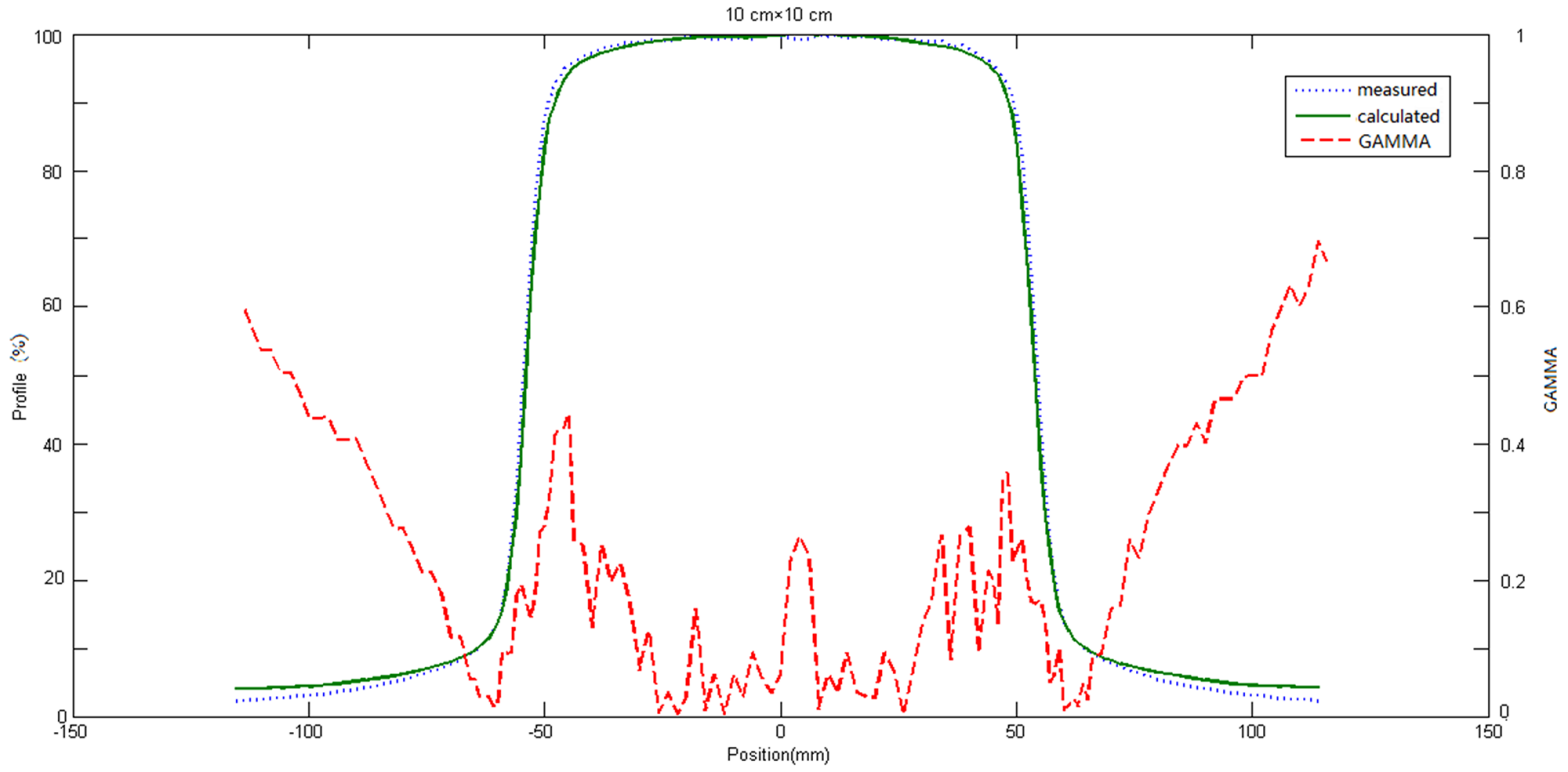
Comparison between OAR of measured radiation field in three-dimensional water tank and reconstructed value of the physical model in 10 cm × 10 cm radiation field The green solid line represented calculated values of physical model, the blue dotted line represented measured values in the three-dimensional water tank, the red dotted line represented the distribution of γ value (3mm/3%).

With changing depth, the motion tracks length of original photons change in different location of phantoms in the process of photon-matter interaction, thus the energy spectrum differ according to different depth and off-axis location [22]. To obtain better calculation accuracy, first, Edose system calculated the absorbed doses of a number of single-energy photons (0.5, 1, 2, 3, 4, 5, 6 MeV) using convolution kernel, and obtained the dose distribution for each photon with certain energy. Then weighted superposition was done according to the accelerator beam energy spectrum to obtain the complete 3D dose distribution. To obtain the ultimate physical parameter values, first, the proportion of photons with each spectrum and the main parameters of physical model were fitted, and then differences between the fitting results and the measured results by the 3D water tank were compared repeatedly to get the optimal parameters for the model. The compared data include PDD, dose profile at corresponding depth and output factors. Results of comparison for dose profiles, PDD and output factors between results of EPID calculation and scanning results of 3D water tank were shown in Figure 1, Figure 2 and Figure 3, respectively. Among them, all differences under portal were not more than 2%; for dose profiles, when the off-axis distance was less than 5 cm, difference between them was less than 1% in the radiation field. The difference between them would be increased, but the difference was less than 3% when the off-axis distance was more than 5 cm. Weight given to photons with different energy and the final physical parameters were shown in Tables 1 and 2, respectively.

**Table 1:** Energy spectrum for accelerator

Energy(Mev)	0.500	1.500	2.500	3.500	4.500	5.500	6.000
Weight	0.024	0.395	0.328	0.126	0.048	0.020	0.001

**Table 2:** Optimized physical model parameters of Edose system

Parameter	c_1_	μ_1_	μ_2_	μ_3_	c_r_	ε	δ
Value	0.00016	14.300	0.310	9.500	-0.025	9.000	3.000

### Tested results of Edose system in clinical applications

#### Tested results of point dose

Differences between results obtained by Edose system and ionization chamber were all very small whether in a homogeneous phantom or inhomogeneous phantom. Difference of point doses in homogeneous and inhomogeneous phantoms were (0.12±0.91)% and (0.03±0.85)% respectively. Detailed results were shown in Table 3.

**Table 3:** Comparison of dose in phantom midpoint

	1	2	3	4	5	Average	SD
σ_*IC_1*_(%)	0.12	1.30	1.04	-0.94	-0.61	0.12	0.91
σ_*IC_2*_(%)	-0.65	0.14	0.66	1.00	-1.01	0.03	0.85

#### Tested results of 2D-plane dose

Table 4 showed that the comparison between 2D dose distribution reconstructed by Edose system and that measured by dosemitric films. There was very little difference between them. γ pass rate was (96.74 ± 0.89)% under the standard of 3mm/3%; γ pass rate was (99.49 ± 0.20)% under the standard of 3mm/5%.

**Table 4:** Comparison of plane dose in inhomogeneous phantom (cross section through the center)

No.	γ_*f*_(%)	
	3mm&3%	3mm&5%
1	96.80	99.58
2	96.14	99.38
3	97.85	99.46
4	95.61	99.25
5	97.28	99.78
Average	96.74	99.49
SD	0.89	0.20

#### Tested results of 3D-volume dose

Table 5 showed the comparison of the 3D dose distributions measured by Edose system and Delta4. γ_*D*_ were (94.18 ± 1.69)% and (98.89 ± 0.27)% under standards of 3mm/3% and 3mm/5%, respectively.

**Table 5:** Comparison between reconstructed dose distribution by Edose system and measured dose distribution by Delta4 in homogeneous phantom

No.	γ_*D*_(%)	
	3mm&3%	3mm&5%
1	94.12	98.73
2	94.54	99.13
3	93.12	98.81
4	96.79	99.20
5	92.35	98.57
Average	94.18	98.89
SD	1.69	0.27

### Preliminary clinical application of *in vivo* dose monitoring by Edose system

#### Tested results of IMRT

As seen in Table 6, the variation of absolute dose at the center of the target volume and γ pass rate within the entire outer contour for the selected 12 IMRT plans were analyzed. Results showed that the absolute dose at the center of the target volume had good concordance and the dose difference was (0.75±1.53)%. The γ pass rate was (96.40 ± 1.47)% under standard of 3mm/5%. Using more strict standard of 3mm/3%, the γ pass rate declined about 7% to (89.11 ± 3.24)%.

**Table 6:** Results of *in vivo* 3D monitoring system for 12 patients received IMRT

No.	Deviation of dose in target volume center(%)	3mm&3%(%)	3mm&5%(%)
1	-1.19	88.15	96.60
2	1.28	87.52	95.45
3	-1.82	94.88	98.03
4	0.64	91.31	98.34
5	1.87	92.77	96.61
6	0.53	88.75	96.65
7	1.26	88.63	97.02
8	0.34	91.33	97.42
9	2.58	83.00	92.67
10	2.25	90.51	96.33
11	-1.35	86.52	96.41
12	2.57	86.02	95.37
Average	0.75	89.11	96.40
SD	1.53	3.24	1.47

**Figure 4:**
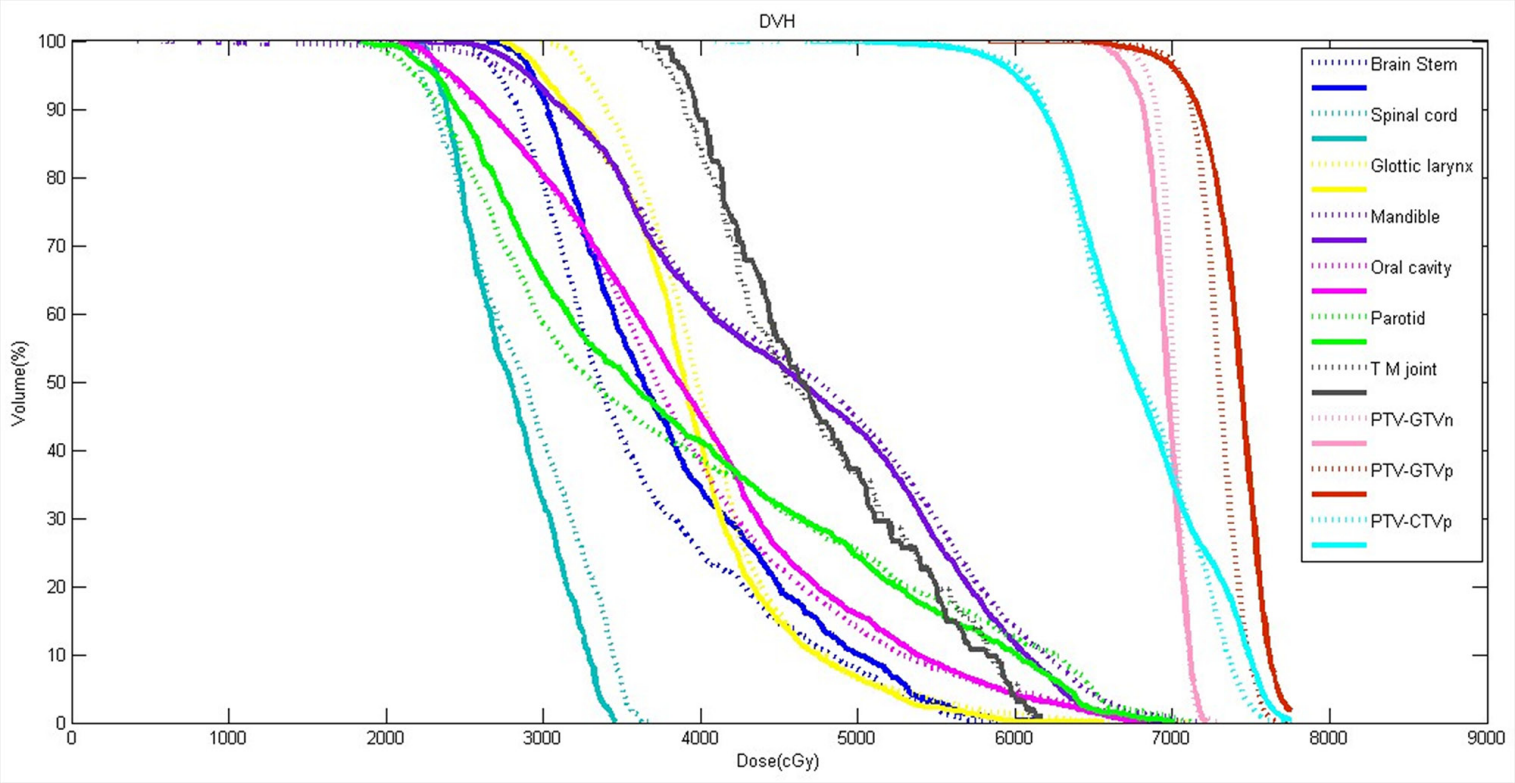
Comparison of DVH curves for a NPC patient The dotted line represented calculated results of TPS, and the solid line represented reconstructed results of *in vivo* three-dimensional dose monitoring system.

Comparison of DVH parameters for all IMRT plans obtained by the Edose and TPS were shown in Table 7. Specially, the results of one NPC IMRT plan were selected and shown in Figure 4. For target volume, the dose differences of D_98%_, D_95%_, D_50%_, D_2%_ for PTV_GTVp were (-2.05 ± 2.92)%, (-0.83 ± 1.96)%, (1.49 ± 1.57)% and (2.85 ± 2.14)%, respectively. Accordingly, the corresponding differences for PTV_GTVn were (-2.46 ± 1.48)%, (-2.90 ± 1.29)%, (-2.10 ± 1.45)% and (0.91 ± 2.61)% and for PTV_CTV were (-2.62 ± 1.40)%, (-2.30 ± 1.16)%, (-1.03 ± 0.87)% and (2.43±2.15)%, respectively. Obviously, the dose differences (for D_98%_, D_95%_, D_50%_, D_2%_) in target volume were all within 3%. However, for brainstem and spinal cord, the the dose differences of D_max_ were (3.31 ± 1.25)% and (-3.90 ± 0.84)%, respectively. For other organs at risk such as Mandible, Parotid, Oral cavity and Glottis larynx, the dose differences were all within 3%.

**Table 7:** Compared results of DVH parameters for 12 IMRT plans

Targets and OARs	D_*98%*_(%)	D_*95%*_(%)	D_*50%*_(%)	D_*2%*_(%)	D_*max*_(%)	D_*mean*_(%)
PTV_GTV_p_	-2.05±2.92	-0.83±1.96	1.49±1.57	2.85±2.14		
PTV_GTV_n_	-2.46±1.48	-2.90±1.29	-2.10±1.45	0.91±2.61		
PTV_CTV_p_	-2.62±1.40	-2.30±1.16	-1.03±0.87	2.43±2.15		
Brain stem					3.31±1.25	
Spinal cord					-3.90±0.84	
T M joint					-1.69±1.50	
Mandible					-1.74±1.82	
Parotid						-1.47±4.50
Oral cavity						2.57±1.33
Glottis larynx						-2.39±0.94

#### Tested results of VMAT

As seen in Table 8, the variation of absolute dose at the center of the target volume and γ pass rate within the entire outer contour for the selected 12 IMRT plans were analyzed. Results showed that the γ pass rate was (97.92 ± 1.38)% under standard of 3mm/5%. Using more strict standard of 3mm/3%, the γ pass rate declined about 5% to (93.04 ± 2.62)%.

**Table 8:** Results of *in vivo* 3D monitoring system for 12 patients received VMAT

No.	3mm&3%(%)	3mm&5%(%)
1	90.57	97.04
2	91.10	97.66
3	95.91	99.30
4	90.29	94.64
5	91.08	98.16
6	94.75	98.47
7	96.69	98.67
8	95.45	99.52
9	88.68	96.06
10	96.19	99.16
11	93.93	98.83
12	91.84	97.52
Average	93.04	97.92
SD	2.62	1.38

## DISCUSSION

This study showed that *in vivo* dose monitoring system based on EPID could monitor the actual dose difference during the radiotherapy. Combined with pre-treatment dose verification or used alone, it might improve the safety and the quality of radiotherapy effectively. However, before putting this system into clinical application, extensive testing for such system is required, such as the optimization of physical model parameters and the measurement procedures.

In the present work, after the physical model parameters were optimized, square calculated results were compared with the measured data by 3D water tank. The differences of output factors were less than 2% in different radiation fields. The maximum difference of PDD curves in the central axis was no more than 2.5%, but the difference became larger with field size increased. These differences might be owing to the fact that the PDD curve of Edose system physical model is obtained by adjusting the proportion of photons with different energy spectrum, without accounting for the electron contamination in actual fitting. By comparing of the results of off-axis curve in depth of 10cm underwater, we found there were good concordance between results calculated from Edose system and results from actual measurement for radiation fields within the radiation field, including the penumbra. Within the radiation field, the differences were less than 1% when the off-axis distance was within the range of 5 cm. However, the difference increased slightly when off-axis distance was more than 5 cm, and yet the difference was still less than 3%. Furthermore, the difference increased significantly when beyond the range of the radiation field. The difference was mainly due to the “over-response to photons of lower energy” phenomenon of EPID [23]. As a result, the dose response of EPID detector was significantly dependent on the off-axis position as well as the thickness of the phantom or patient in the beam. In general, IMRT dose verification focused mainly on the dose change within the field and the penumbra. Our results showed that dose differences within the field or in the penumbra area were both in a reasonable range, so the Edose system could be used in clinical tests. However, further amendments of “over-response to photons of lower energy” phenomenon would help to improve the accuracy of the system in *in vivo* dose reconstruction.

From the testing results of clinical application in phantom, we found that the dose differences at the center point of target volume were less than 2%, whether in homogeneous phantom or in inhomogeneous phantom. Particularly, in homogeneous and inhomogeneous phantom, the gamma pass rates were all more than 90% (3mm/3%) while the gamma pass rate even reached to more than 95% under 3mm/5%. However, when the relatively stricter standard of 2mm/2% was employed, the result decreased significantly, with the gamma pass rate of (78.97 ± 3.38)% and (74.63 ± 4.65)% in homogeneous and inhomogeneous phantom, respectively. Moreover, the gamma pass rate in inhomogeneous phantom degraded notably than that in homogeneous phantom. The possible reasons are as follows. 1) With the stricter standard, the measurement errors existing in the film dosimetry had a greater effect on the result. 2) In inhomogeneous phantom, the calculation accuracy may be compromised in the three-dimensional reconstruction dose of Edose system.

In terms of the patients’ *in vivo* dose monitoring, results showed that the dose differences at the center of target volume were also less than 3%, but the 3D gamma pass rate was lower than that in phantom and also lower than AAPM’s criterion [24], but it not better correlated with the dosimetric differences actually observed in the DVHs. Comparison of DVH parameters for patients showed that the dose differences of all targets volume (for D_98%_, D_95%_, D_50%_, D_2%_) and mostly organs at risk (for D_max_ or D_mean_) were within 3%. For brainstem and spinal cord, the dose differences revealed to be higher, ranging from 3% to 5%. This may be due to the dose calculation method and the DVH statistical for organs of small volume by different systems. Our preliminary experiences for 3D *in vivo* dose monitoring also showed that *in vivo* dose verification for patients was much more complicated than that in phantom. Therefore, for the next step towards clinical application, we would choose the Gamma pass rate 85%(3mm/3% as standard), or adopt less strict standard such as gamma pass rate of 90% with the standard of 3mm/5%.

The dose difference during radiotherapy mainly come from two aspects [2]. First, dose difference might be generated by the operation of the accelerator; it might also stem from the differences among different three-dimensional dosing algorithms, particularly form the dose calculation for inhomogeneous tissues [29, 25-26]. Fully understanding the causes of the dose difference would help us to further improve the accuracy of radiotherapy. Unfortunately, the Edose system could not undertake the independent dose calculation at present, and thus the specific factors causing the dose difference are difficult to be identified and distinguished. Therefore, for better quality assurances and detailed analysis about the cause of errors, it is necessary to implement a series of checking, such as independent testing, pre-treatment dose verification, and even the correctness checking for execution file of the reference machine and so on.

In addition, in the preliminary research of clinical application for Edose system, *in vivo* dose monitoring was mainly used in the first treatment of patients. The dose distribution was calculated and reconstructed based on the patient’s plan CT. To monitor the *in vivo* dose at the whole course of the radiation treatment, the changes of patients’ position or the target volume should be taken into account because the simulation or plan CT images can not reflect the real-time anatomy information of the patients. In fact, there may be a large differences between the *in vivo* dose monitoring results and the actual situation [27]. Therefore, it is necessary to use a concurrent treatment image of the patient (CBCT, on-rail CT, etc.) instead of the plan CT for real-time dose reconstruction. On the basis of this, the slight changes of target volume and organs at risk as well as the machine’s error could be reflected simultaneously. Thus, physicians can easily identify the reasons of dose difference for patients, and determine whether the patients need a adjusted or even a new treatment plan.

Currently, EPID has been widely used in the pre-treatment dose verification of IMRT, and also be applied gradually to *in vivo* dose monitoring. However, compared to pre-treatment dose verification, the “over response” feature of EPID detector seems to be a more detrimental factor affecting the accuracy of *in vivo* dose monitoring [23]. The patient’s body thickness results in spectrum changes of the photon rays, which affects the accuracy of EPID dosimetrical measurements. Moreover, scattered radiation from the human body, with low-energy, may also affect the accuracy of EPID measurements. Given those uncertainties, more extensive clinical research and experiences are needed to examine and improve he accuracy of the Edose system. This is our top research priority for the next step.

## MATERIALS AND METHODS

### Equipment and image acquisition

The 6MV photon beams of the Unique linear accelerator system (Varian Medical Systems, Palo Alto, CA, USA) were used for all measurements. The accelerator is equipped with an aS1000 EPID which had a sensitive area of 40 cm (cross-plane) × 30 cm (in-plane) in size, and an effective pixel size of 0.039 cm × 0.039 cm. The source-to-detector distance was set to 140 cm (SDD=140 cm) during the *in vivo* dose monitoring. Image acquisition was performed in integrated mode with a Varian IAS system, and offset correction; gain correction and pixel correction were performed for each image. Because of the robotic arm that is located directly beneath the sensitive area of the Varian EPID, the backscatter from the arm can have a deleterious effect when the EPID is used for dosimetric purposes [28, 29]. So the backscatter correction should also be amended.

In this study, the treatment planning system (TPS) was Eclipse 10.0 (Varian Medical Systems, Palo Alto, CA, USA) with the analytic anisotropic algorithm (AAA), calculating grid for all programs was 2.5mm × 2.5mm × 2.5mm.

Three different types of phantoms were used in the current study, which including: IMRT homogeneous phantom (IBA company, Belgium), Delta4 cylindrical phantom (ScandiDos, Uppsala, Sweden) and inhomogeneous chest phantom (THORAX 002LFC, CIRS, USA); Measurement tools used included: Finger-type ionization chamber (Farmer 2571, NE, UK), Delta4 semiconductor matrix (ScandiDos, Uppsala, Sweden) and dose film (EBT3, ISP, USA). The dosimetric films were scanned by EPSON 10000XL scanner, and dose distributions from these films were analyzed by the QALAB system (company of Raydose, China).

### Introduction of physical model for Edose system

**Figure 5:**
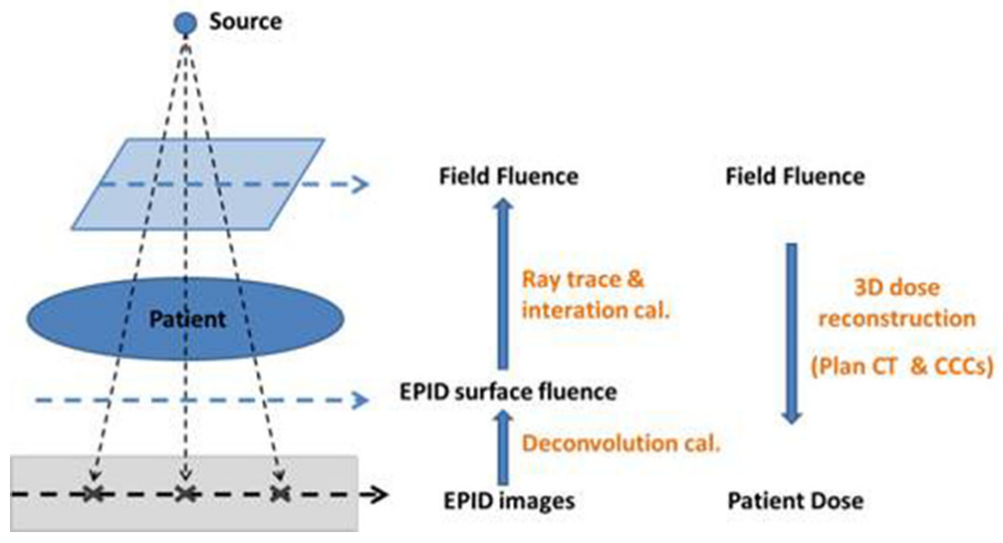
3D dose reconstruction principle of Edose system First, the surface fluence of EPID was calculated using deconvolution method. Then the fluence of the field was calculate using ray tracing and iterative calculation method. Finally, 3D dose distribution was reconstructed on patient’s Plan CT using CCCs algorithms based on GPU.

In Edose system, the reconstruction process of 3D dose distribution for patients was divided mainly into two steps: First, Backstepping the fluence maps were draw based on the measurement results of EPID [30, 31]. Then using CCCs algorithm, the 3D dose distribution for actual patients in the planning CT was reconstructed based on graphic processing unit (GPU) [31]. Detailed processes were shown in Figure 5.

In the system, the physical model optimization was done by accounting for seven physical parameters: μ_1_, μ_2_, μ_3_, c_1_, ε, δ, c_r_. Among them, μ_1_ and μ_2_ were the physical parameters describing nuclear scattering of EPID, μ_1_ described the rapid drop portion origin near the central part, μ_2_ described the trailing slowly declining portion in the distance, c_1_ reflected the proportion of μ_1_ and μ_2_, and it is relevant to phantom depth in reconstruction;μ_3_ was a physical parameter describing the fuzzy convolution kernel of EPID, it is used to correct the penumbra; ε, δ, c_r_ were mainly used to do the comparison and adjustment for relative dose after reconstruction, and the adjustment was compiled through comparing the off-axis ratio of field curves (OARs).

### Physical modeling and *in vivo* dose monitoring processes of Edose system

**Figure 6:**
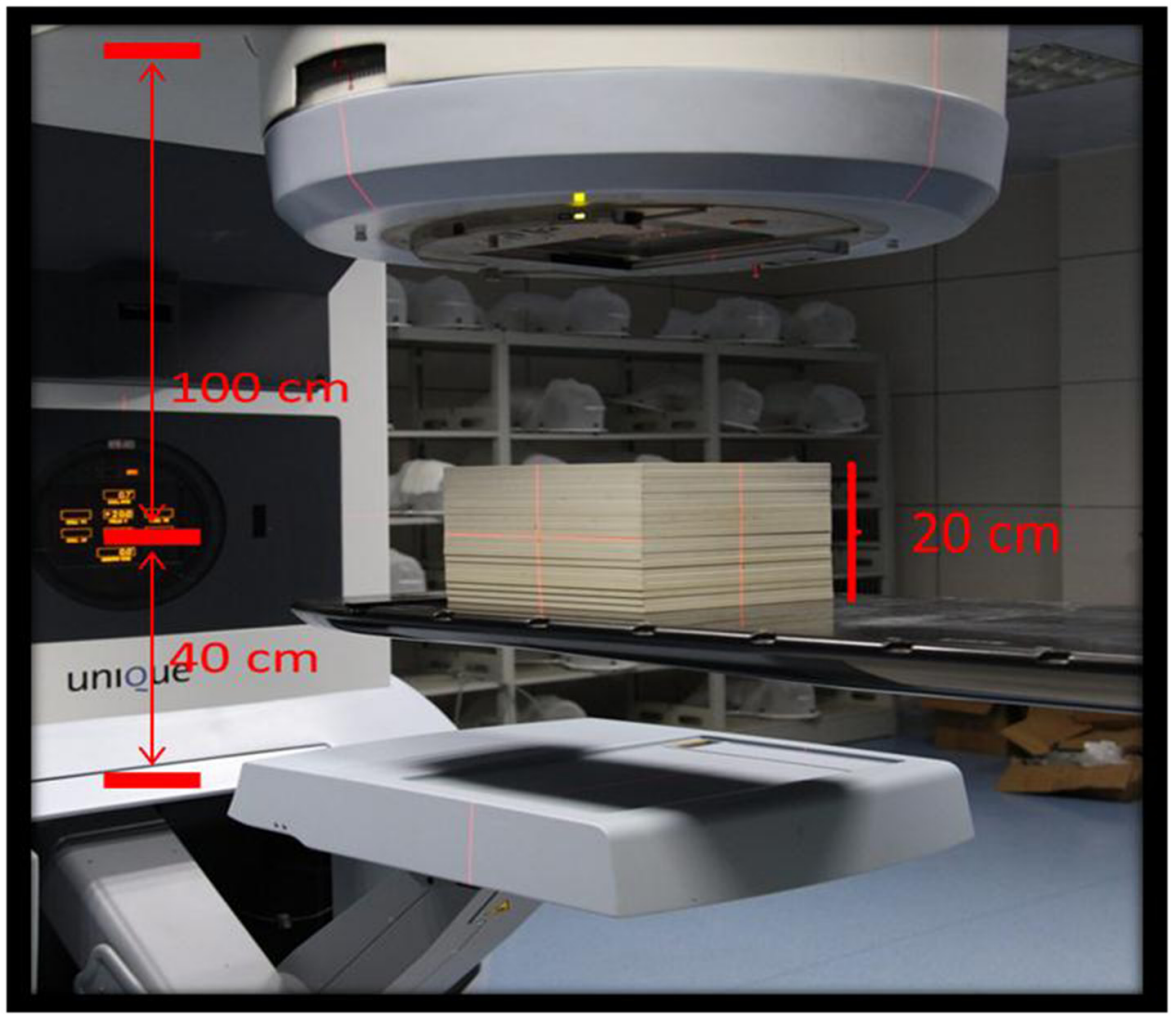
Schematic of phantom positioning in the data collection for modeling Solid water thickness was 20cm, SSD=90cm, source-to- EPID detector distance was140cm.

The 3D dose reconstruction of Edose system is based on the algorithm model, therefore, physical modeling is required in accordance with the conditions before application. The following data are required for physical modeling:

A series of EPID portal images of square fields(3 cm×3 cm, 5 cm×5 cm, 10 cm×10 cm, 15 cm×15 cm, 20 cm×20 cm and the maximum range), obtained at 0 gantry angle within a water phantom with dimension of 30 × 30 × 20cm3 (length × width × height) interposed in the beam. As shown in Figure 6, the measurement conditions were: Source-to-surface distance was 90 cm (SSD = 90 cm), source-to-detector distance was 140 cm (SDD = 140cm), and the monitor units were 100 MU.

A typical data set in water tank for the modeling of the accelerator: the percentage depth dose (PDDs), the total scatter factors (S_c, p_) and OARs of square fields(3 cm×3 cm, 5 cm×5 cm, 10 cm×10 cm, 15 cm×15 cm, 20 cm×20 cm). By comparing the calculated results of Edose system and the measurement results in water tank, these data are mainly used to correct and adjust the physical parameters for the optimization of the physical model.

Absolute calibration: By scaling the central point dose of the reference field (10 cm × 10 cm), measurements were done through the ionization chamber (D_*Ion*_) and EPID (D_*Epid*_). The scale factor of absolute dose was recorded as C_*ad*_, C_*ad*_ = D_Epid_ / D_*Ion*_.

**Figure 7:**
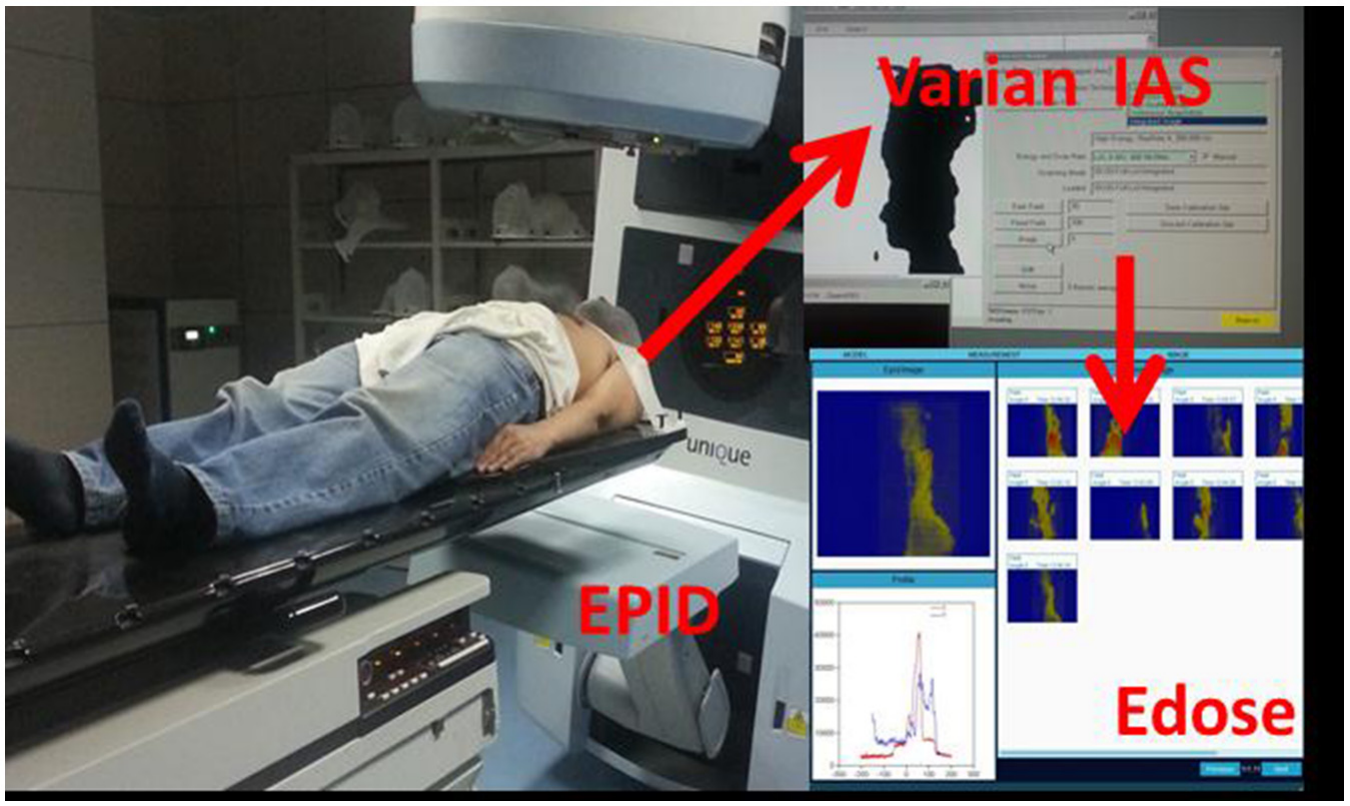
EPID image acquisition The image acquisition was performed with IAS3 monitor of Varian. EPID images were captured using the integrated image mode in the treatment; EPID images and logfiles of the actual irradiation angle were transferred to the Edose system automatically.

**Figure 8:**
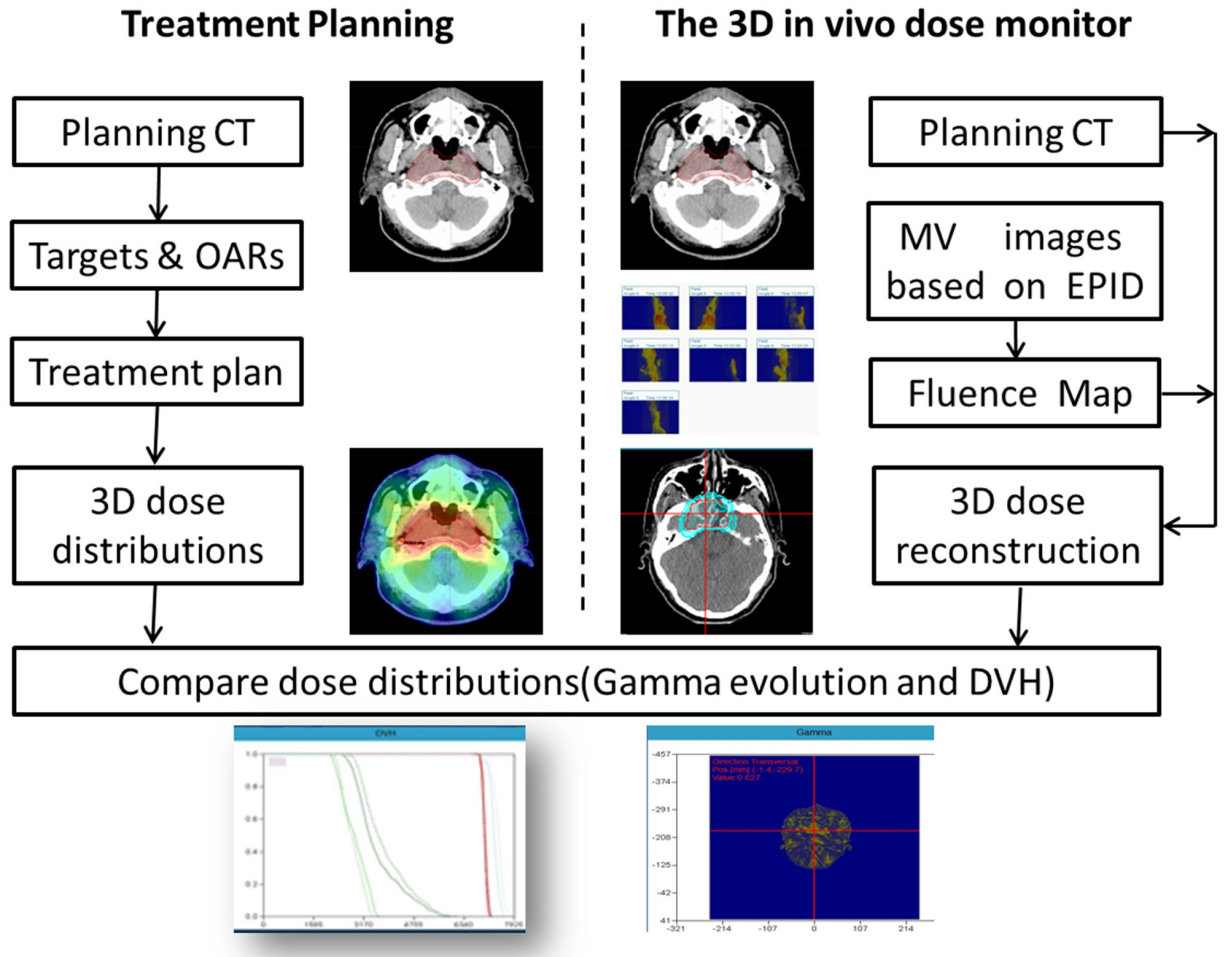
Work flow of 3D *in vivo* dose verification with the Edose system The left side of the figure shows the procedure of radiation treatment planning, and the dose calculated by TPS is used as the reference dose when compared with the EDose. The right side shows the dose reconstruction procedure of EDose system. First, the EPID images are used for calculating fluence of the fields, then, dose is reconstructed with CCC algorithm on Planning CT images of the patients. The comparison between 3D reconstruction dose and TPS results by EDose system is carried out by the method of Gamma analysis and DVH.

*In vivo* dose monitoring process of Edose system was shown in Figure 7: First, the planning data of the patients or phantom (CT images, structures, RTPlan file, RTdose file) was imported into Edose system. Then, the dose distributions of various portal angels were acquired by EPID during radiotherapy. After the treatment, the images and logfile documents of actual irradiation angles were exported to Edose system automatically. Then the angle matching and 3D dose reconstruction was performed as well. The workflow of Edose was shown in Figure 8.

### Clinical application test of Edose system

#### Point dose verification

Five IMRT plans selected were transferred to an IMRT homogeneous phantom and a chest inhomogeneous phantom, and plans data were exported to Edose system. The absolute dose at the center of target volume was obtained through Edose system and ionization chambers during the dose delivery. Dose reconstructed by Edose system was marked as D_*E*_; dose measured by ionization chambers was marked as D_*IC*_. Difference between these two doses, σ_*IC*_, was calculated by the formula (1). Results from homogeneous and inhomogeneous phantoms were noted as σ_*IC_1*_ and σ_*IC_2*_, respectively.

$$\sigma_{IC}=\left[\left({\text{D}}_{E}-{\text{D}}_{IC}\right)/{\text{D}}_{IC}\right]\times 100\%$$ (1)

#### 2D-plane dose verification:

The five IMRT plans as mentioned in section Point dose verification were transferred to chest inhomogeneous phantom, and plans data were exported to Edose system. The two-dimensional dose distribution through the center of the cross section of phantoms was obtained by the Edose system and measured by dosimetric film (EBT3, ISP, USA) during the dose delivery. Subsequently, dose distributions reconstructed by Edose system and measured by the dosimetric film were analyzed by Global Gamma analysis of relative dose (the central dose as normalization point) [32-34]. Gamma pass rates were recorded as γ_*f*_.

#### 3D-volume dose verification

Similarly, the same five IMRT plans were transferred to cylindrical phantoms of Delta4, and plans data were exported to Edose system. The 3D dose distribution of the phantoms was obtained by the Edose system and by the Delta4 during the dose delivery. Dose distributions were then analyzed by three-dimensional Gamma analysis. Gamma pass rates were recorded as γ_*D*_.

### Preliminary analysis of clinical utilization of Edose system

#### IMRT (sliding window)

The clinical applications of the Edose system were examined preliminarily in 12 patients with radiotherapy for the head and neck region. *In vivo* dose monitoring was done by Edose system during the patients’ first treatment. Patients’ positions were all verified with EPID before the dose delivery. The corresponding results obtained by Edose and TPS were compared, including the difference of absolute dose at the center of target volume, the gamma pass rates with the standard of 3mm/3% and 3mm/5%, as well as the DVH of target volume and organs at risk. Dose parameters of target volume were compared including the difference of D_98%_, D_95%_, D_50%_, D_2%_ and other parameters. Comparisons were made for differences of Dmax and other doses in organs at risk such as spinal cord and brain stem.

#### VMAT (volumetric modulated arc therapy)

The clinical applications were examined preliminarily in 12 patients with radiotherapy for various anatomical sites (head & neck, thorax and abdomen). A Synergy linear accelerator system (Elekta AB, Stockholm, Sweden) with an iViewGT EPID (Elekta AB, Stockholm, Sweden) was employed in this test. The source-to-detector distance was set to 160 cm (SDD=140 cm) during the *in vivo* dose monitoring. *In vivo* dose monitoring was done by Edose system during the patients’ first treatment. Patients’ positions were all verified with CBCT (Cone Beam CT) before the dose delivery. The corresponding results obtained by Edose and TPS were compared, including the difference of absolute dose at the center of target volume, the gamma pass rates with the standard of 3mm/3% and 3mm/5%.

## CONCLUSIONS

In this study, we carried out both the preclinical and preliminary clinical testing of the Edose system. The system can not only improve the safety and quality assurance of radiotherapy but also provide additional information for further Adaptive Radiation Therapy and Dose-guided Radiation therapy. Nevertheless, for clinical application, more studies for *in vivo* dose monitoring remain to be performed to improve the accuracy of the system and to explore better workflows.

### Ethics approval and consent to participate

In this study a new quality assurance (QA) tool to verify the accuracy of RT dose delivery based on EPID was evaluated. This work did not affect patient’s treatment. Digital information, already routinely acquired for patient-specific QA, was used, i.e., for patients no additional investigations or measurements were required. For these reasons, according to the Ethics Committee of our hospital, no ethical approval was required for this study. Providing that all patient-related information was anonymized prior to presentation and publication, informed consent from the patients was not needed either.

### Consent for publication

Written consent was obtained from the patient for publication.

### Availability of data and materials

The datasets supporting the conclusions of this article are included within the article.

## Abbreviations

EPID: Electronic portal imaging device
IMRT: Intensity-modulated radiation therapy
SBRT: Stereotactic body radiation therapy
MLC: Multi-leaf collimator
GPU: Graphic processing unit
CCCS: Collapsed-cone convolution/superposition
PBC: Pencil-beam calculation
OAR: Off-axis ratio
TPS: Treatment planning system
DVH: Dose-volume histogram
CBCT: Cone beam computed tomography
